# An efficient neural network LEACH protocol to extended lifetime of wireless sensor networks

**DOI:** 10.1038/s41598-024-75904-1

**Published:** 2024-11-06

**Authors:** Hamdy H. El-Sayed, Elham M. Abd-Elgaber, E. A. Zanaty, Faisal S. Alsubaei, Abdulaleem Ali Almazroi, Samy S. Bakheet

**Affiliations:** 1 Department of Computer Science, Faculty of Computers and Artificial Intelligence, Sohag University, Sohag, 82524 Egypt; 2 Department of Cybersecurity, College of Computer Science and Engineering, University of Jeddah, Jeddah, Saudi Arabia; 3 Department of Information Technology, Faculty of Computing and Information Technology in Rabigh, King Abdulaziz University, 21911 Rabigh, Saudi Arabia; 4 College of Computer Engineering and Science, Prince Sattam Bin Abdulaziz University, Al-Kharj, Saudi Arabia

**Keywords:** WSNs, Neural networks, Energy hole, LEACH, ILEACH, NN_ILEACH, Energy science and technology, Engineering

## Abstract

This paper presents NN_ILEACH, a novel neural network-based routing protocol designed to enhance the energy efficiency and longevity of Wireless Sensor Networks (WSNs). By integrating the Energy Hole Removing Mechanism (EHORM) with a sophisticated neural network for cluster head selection, NN_ILEACH effectively addresses the energy depletion challenges associated with traditional protocols like LEACH and ILEACH. Our extensive simulations demonstrate that NN_ILEACH significantly outperforms these classical protocols. Specifically, NN_ILEACH extends the network lifetime to an impressive 11,361 rounds, compared to only 505 rounds achieved by LEACH under identical conditions—representing a more than 20-fold improvement. Additionally, NN_ILEACH achieves a 30% increase in throughput and a 25% enhancement in packet delivery ratio, while reducing overall energy consumption by 40%. These results underscore the protocol’s potential to optimize energy usage and maintain network stability, paving the way for more resilient IoT systems in dynamic environments. Future work will explore further integration of machine learning techniques to enhance adaptability and performance in WSNs.

## Introduction

The Internet of Things (IoT) refers to the capability of connecting devices that can be monitored or controlled via the Internet, anytime and anywhere. Wireless technology serves as an effective medium for achieving this connectivity across a wide range of applications. Wireless Sensor Networks (WSNs) act as a virtual interface, bridging the real and digital worlds by transmitting sensed data from the physical environment to the Internet. This function makes WSNs the “ears” and “eyes” of IoT^1^.

IoT systems facilitate communication among numerous devices across various settings, including homes, offices, agriculture, industries, transportation, and even battlefields, which significantly increases infrastructure demands. WSNs are essential to these systems, consisting of multiple wireless sensing nodes that gather various physical data and send it to a centralized base station or sink for processing and analysis.

As IoT expands, the number of sensing nodes will grow exponentially. This increase presents challenges, such as the need for more communication frequency spectrum, enhanced data security, and higher energy requirements for node operation. The primary requirement for sensing nodes is energy; without it, they cannot function. Additionally, since WSN nodes are often small and deployed in remote locations, traditional methods of recharging or replacing batteries are not feasible^2^.

With a high density of sensors but limited battery power, energy conservation becomes critical in WSNs. Routing protocols play a significant role in energy efficiency, particularly when energy usage is prioritized in their design. Cluster-based routing protocols are recognized for their effectiveness in conserving energy, thereby extending network lifetimes. Clusters consist of sensor nodes, with a special node known as the cluster head (CH) that gathers and transmits data from ordinary nodes to the base station. This clustering approach enhances scalability and reduces radio transmissions.

The selection of the CH is crucial as it directly affects network performance. The ideal CH should be chosen based on factors such as energy level and location to maximize system stability and overall network longevity. Consequently, researchers focus on improving WSN protocols to enhance lifespan and reduce energy consumption^3^.

In addition, security poses a significant challenge in Wireless Sensor Networks (WSNs) due to their unique characteristics and operational environments. The broadcast nature of wireless communication makes WSNs particularly vulnerable to various attacks, such as eavesdropping and Denial of Service (DoS). Additionally, the limited resources of sensor nodes, including energy, memory, and processing power, restrict the implementation of robust security protocols. Many existing security mechanisms are not fully applicable to WSNs, necessitating tailored solutions that balance security and performance. Therefore, ongoing research is crucial to address these vulnerabilities and develop effective security frameworks for WSN applications^4^. While our proposed NN_ILEACH protocol primarily focuses on energy efficiency in WSNs, we acknowledge the significance of integrating security mechanisms. Our approach enhances the network’s longevity and stability, which indirectly supports the resilience of the network against attacks. Future work will explore how energy-efficient strategies can be adapted to include security measures for attack detection without compromising energy conservation.

In this paper, we propose a novel mechanism that utilizes neural networks to improve cluster head selection and an Energy Hole Removing Mechanism (EHORM) to optimize energy use. Machine learning, which involves algorithms that create predictive models, can be categorized into supervised, unsupervised, and reinforcement learning. By employing machine learning, systems can analyze input data to make predictions^5,6^. Our approach utilizes supervised learning, where a model is built using predefined inputs and known outputs to capture the relationships within the system. We apply neural networks to enhance the ILEACH protocol.

These networks, inspired by simplified brain models, consist of interconnected neurons linked by weighted connections. Our proposed algorithm aims to prolong the network lifespan and conserve energy. Preliminary results show that the proposed method maintains node functionality for 11,361 rounds with an initial energy of 0.5 J/node, compared to just 505 rounds for the LEACH protocol under the same conditions. This demonstrates that NN_ILEACH significantly extends network longevity, achieving over 20 times the lifespan of competing protocols^7^.

## The novel contributions

This study makes the following novel contributions:

1. Novel Routing Protocol: We provide NN_ILEACH, a new neural network-based routing protocol that improves energy efficiency and extends the lifetime of WSNs by optimizing cluster head selection.
2. Machine Learning Integration with the ILEACH Protocol: Our approach takes advantage of supervised learning techniques, using historical data to train a neural network that dynamically selects the best cluster heads based on energy levels, addressing the limitations of traditional random selection methods.
3. Energy Hole Removing Mechanism (EHORM): We have incorporated the Energy Hole Removing Mechanism (EHORM) into the NN_ILEACH protocol. This technique effectively addresses energy depletion by balancing energy use across the network, reducing premature node failures, and improving overall network stability.
4. Performance Improvements: Extensive simulations show that NN_ILEACH surpasses previous protocols, with an amazing 11,361 rounds of operation compared to only 505 rounds for classic LEACH. Furthermore, NN_ILEACH increases throughput by 30% and improves the packet delivery ratio by 25% while consuming 40% less energy.
5. Framework for Future Research: Our findings pave the way for future research aimed at incorporating advanced machine learning techniques into WSN protocols, hence boosting flexibility and performance in dynamic contexts.
6. Applicability: The idea of our protocol can be used with any new wireless sensor network protocol.

The rest of this paper’s sections will be as follows: In section “Related work”, we will show related work. In section “Wireless sensor networks (WSNs)”, we will show wireless sensor networks. In section “Integration with EHORM”, we will show our proposed scheme. In section “Applicability: The idea of our protocol can be used with any new wireless sensor network protocol.”, we will represent simulation results and discussion. In section “Statistics and analysis”, we will show statistics and analysis. In section “Conclusion”, we will represent the conclusion, and in section “Future work” will be the future work.

## Related work

Several research studies have been conducted in the field of Wireless Sensor Networks (WSNs) to address the challenges of energy efficiency and cluster head selection. In this section, we discuss some relevant works that have tackled similar problems and highlight the similarities and differences with the proposed scheme.

One notable work is the LEACH protocol proposed by Heinzelman et al.^8^. LEACH utilizes a randomized approach for cluster head selection in each round, but it does not consider the energy levels of cluster heads. This random selection can lead to energy imbalances within clusters and potential energy depletion of cluster heads, resulting in the loss of connectivity with the base station. In contrast, our proposed scheme, NN_ILEACH, incorporates neural networks to enhance cluster head selection. By training the neural network using historical data, we aim to make more informed decisions based on energy levels, thus improving the selection process and mitigating energy imbalances.

Another relevant work is the ILEACH protocol proposed by Youssef et al.^9^. ILEACH incorporates an energy threshold (Eth) to mitigate the selection of low-energy cluster heads. However, it still relies on random selection, which may not guarantee optimal cluster head choices. In our proposed scheme, we build upon the concepts of LEACH and ILEACH and introduce a novel approach that leverages neural networks for cluster head selection. This approach ensures that the most suitable cluster heads are chosen, addressing the limitations of random selection methods.

Furthermore, Zhang et al. proposed a neural network-based approach for cluster head selection in their work^10^. Their approach demonstrated improved energy efficiency and network performance compared to traditional methods. While their work shares similarities with our proposed scheme in terms of using neural networks, our scheme extends the analysis by integrating the Energy-efficient Hole Removing Mechanism (E-HORM). This mechanism helps close energy gaps within the network, further improving energy consumption and prolonging the network’s lifetime.

Additionally, other works have explored various aspects of cluster head selection in WSNs. For instance, Li et al. proposed a genetic algorithm-based approach for cluster head selection^11^, focusing on optimizing network lifetime. Gupta et al. introduced a fuzzy logic-based approach for cluster head selection, aiming to balance energy consumption among nodes^12^. These works highlight alternative methods for cluster head selection, but they differ from our proposed scheme in terms of the specific techniques employed.

Devi et al.^13^ several studies have addressed the energy-efficient cluster head selection challenge in LEACH protocols. Devi Rita et al. proposed a “Node Prioritization Based Load Balancing Approach” that selects cluster heads based on factors like energy level, distance to other nodes, and centrality in the network. This approach aims to distribute the load evenly among cluster heads, extending the network’s lifetime. While their work shares the goal of improving LEACH’s efficiency, it differs from ours in its selection method.

This paper utilizes a modified version of LEACH using neural networks for the process of cluster head selection and the EHORM mechanism for reducing energy consumption.

As an energy-efficient CH selection, K.Amirthalingam et al.^14^ propose an improved leach. The parameters are energy and distance. The probability function is modified with the distance and energy metrics to select the most energy-efficient cluster head to transmit data.

While their approach aims to balance cluster load and improve energy efficiency, it differs from NN_ILEACH in several key aspects:

Cluster head selection method: Improved LEACH uses pre-defined thresholds and weighting factors, whereas NN_ILEACH leverages a machine learning approach with a neural network for dynamic and data-driven selection.

Energy management: Improved LEACH primarily focuses on load balancing, while NN_ILEACH additionally incorporates the EHORM mechanism for adaptive power control and targeted energy conservation.

Performance metrics: While both works likely evaluate network lifetime and energy consumption, NN_ILEACH also considers additional metrics like throughput, packet delivery ratio, and the number of nodes reaching the base station.

Zaho et al.^15^ proposed a modified LEACH by improving the selecting cluster heads’ equation and considering the dynamic change of energy. A vice cluster head for each cluster was established during the communication process. That aims to reduce the consumed energy during the reclustering and to extend the time of being in a steady-state phase.

Daanoune et al.^16^ propose an “improved LEACH” protocol that focuses on selecting cluster heads based on remaining energy, balancing cluster sizes, and identifying abandoned nodes for efficient data transmission. Similar to our work, it aims to increase network lifetime by optimizing energy consumption during cluster formation and data aggregation. However, our work differs from it in some aspects, such as the cluster head selection. We leverage neural networks in this process, but they give priority to the remaining energy and the cluster size. They also focus on identifying and handling abandoned nodes in the process of data transmission optimization, but we use the energy hole-removing mechanism to reduce the consumed energy.

Saikia et al.^17^ propose an “improved LEACH” protocol that focuses on three key aspects: Enhanced cluster head selection by incorporating a dynamic threshold based on remaining energy and node density to select cluster heads, aiming for balanced cluster sizes and energy distribution, Adaptive transmission power by adjusting transmission power based on distance to the cluster head, reducing energy consumption for farther nodes, and data aggregation by implementing a two-level aggregation scheme for efficient data forwarding.

While this work shares the goal of improving LEACH’s efficiency with our proposed protocol, key differences exist, such as the fact that NN_ILEACH utilizes a neural network to learn optimal cluster head selection, potentially surpassing rule-based methods in adaptability and accuracy. The EHORM mechanism in our NN_ILEACH goes beyond Saikia et al.’s approach by dynamically adjusting power based on energy levels, potentially achieving more aggressive energy conservation.

Ramesh et al.^18^ proposed a modified k-means algorithm with the LEACH protocol for optimizing the Wireless Sensor Network. The weight of the cluster head is tested and elected in the modified k-means algorithm, and clusters are formed using the Euclidean distance formula. When compared to the existing protocol, the proposed work saves 48.85% of the time. It has also been demonstrated that the proposed work resulted in more successful data transfer to the sink node. The cluster head selection process chooses the most efficient node with the lowest failure rate as the cluster head. The proposed work optimistically balanced the entire network in terms of energy and data transfer success.

While they focus on optimizing the LEACH protocol using machine learning techniques, our NN_ILEACH protocol takes it a step further by proposing a new neural network-based routing algorithm for cluster head selection. By leveraging neural networks, our protocol can make more intelligent and efficient decisions when selecting cluster heads, resulting in improved network performance.

In addition to the neural network-based cluster head selection, the NN_ILEACH protocol incorporates the EHORM (Energy Hole Removing Mechanism). This mechanism helps in reducing the energy consumed by nodes over the network during rounds. By addressing energy holes and optimizing energy consumption, our protocol can effectively prolong the network’s lifetime compared to the approach presented in their paper.

Nabavi et al.^19^ presented a novel optimization approach for clustering wireless sensor networks using the multiobjective genetic algorithm and the gravitational search algorithm. To select the cluster head, a multiobjective genetic algorithm based on reducing intracluster distances and energy consumption of the cluster nodes is used, and nearly optimal routing based on the gravitational search algorithm is used to transfer information between the cluster head nodes and the sink node. The implementation results show that taking into account the capabilities of the multiobjective genetic algorithm and the gravitational search algorithm, the proposed method outperforms the previous methods in terms of energy consumption, efficiency, data delivery rate, and information packet transmission rate.

In our research, we propose the NN_ILEACH protocol, which leverages neural networks for cluster head selection and incorporates EHORM for energy savings. By doing so, our proposed protocol offers several advantages over the approach presented in^19^, such as:

NN_ILEACH utilizes the power of neural networks to make intelligent decisions for cluster head selection. This enables the protocol to adaptively select cluster heads based on various factors, such as node characteristics, network conditions, and energy levels, resulting in improved network performance. In this paper, we just used the energy, and it made a great enhancement in several metrics, as will be mentioned later. The integration of EHORM in NN_ILEACH further enhances energy efficiency by addressing energy holes and reducing energy consumption during rounds. This mechanism ensures a more balanced energy distribution among nodes, leading to a prolonged network lifetime.

Bhola et al.^20^ Proposed an energy-efficient protocol based on LEACH and the optimization genetic algorithm (GA). LEACH is a hierarchical protocol that transforms sensor nodes into cluster heads (CH), who gather and compress data before sending it to the target node. Using its fitness function, the genetic algorithm assists in determining the best route. When GA is used, the energy consumption rate is reduced by up to 17.39% after simulating the code in MATLAB. Finally, a comparison of the proposed work and existing work is performed to determine the proposed work’s efficiency.

In our research, we propose the NN_ILEACH protocol, which incorporates neural networks for cluster head selection and integrates EHORM for energy savings. By leveraging neural networks, our proposed protocol achieves more intelligent and adaptive cluster head selection compared to the genetic algorithm-based approach presented in^20^.

Furthermore, the integration of EHORM in NN_ILEACH contributes to additional energy savings by addressing energy holes and optimizing energy consumption during rounds. This mechanism ensures a more balanced energy distribution and prolongs the network’s lifetime.

Khan et al.^21^ also identifies the flaws in the existing LEACH protocol, proposes a novel methodology for improving the LEACH protocol, and compares it to the basic LEACH approach. It seeks to extend network lifespan by reducing energy consumption, primarily by exploiting the limitations of the LEACH and its related algorithms.

Various algorithms have been proposed to enhance energy efficiency in Internet of Things (IoT) environments. One such significant contribution is the Tunicate Swarm Algorithm-based Optimized Routing Mechanism (TORM) presented by Dogra et al. (2021). This study addresses the energy efficiency of sensor nodes by employing the Tunicate Swarm Algorithm (TSA) for optimal selection of Cluster Head (CH) nodes. TORM utilizes five fitness parameters, including available energy, initial energy level, distance from the sink, inter-cluster distance, and node density, to enhance network sustainability. The simulation results demonstrated that TORM significantly outperformed several state-of-the-art algorithms, improving both the stability period and network lifetime. This work highlights the importance of optimization methods in routing protocols for IoT networks. While TORM excels in network lifetime and stability due to its optimization approach, NN_ILEACH offers advantages in adaptability, dynamic operations, and enhanced data management. These features can make NN_ILEACH a more suitable choice for applications requiring real-time responsiveness and efficient energy utilization^22^.

Verma (2021) presents an innovative approach known as the energy-efficient routing paradigm for resource-constrained IoT-based cognitive smart cities (EI-CSC) in the context of energy-efficient routing in resource-constrained environments. This work utilizes the Sooty tern optimization algorithm (STOA) to enhance the selection of cluster heads, significantly improving the stability period and network lifetime by 83.4% and 107.7%, respectively, compared to existing algorithms such as genetic algorithms and PSO-based hybrid clustering algorithms. Verma’s study emphasizes the critical role of energy management in deploying wireless sensor networks within smart cities, addressing challenges posed by resource limitations in IoT devices. However, while this paradigm effectively addresses energy constraints, it primarily relies on traditional routing strategies that may not fully exploit the adaptive capabilities offered by machine learning. In contrast, our proposed NN_ILEACH protocol integrates a neural network-based approach for cluster head selection, which allows for a more data-driven and context-aware selection process^23^.

The AGRIC protocol stands out as a novel solution that uses artificial intelligence to improve routing efficiency in industrial cyber-physical systems, especially in harsh environments. AGRIC focuses on reducing energy usage while maintaining reliable data transfer, making it suitable for applications that require strong performance under difficult conditions.

Our suggested NN_ILEACH protocol prioritizes energy efficiency and network lifetime, which aligns with AGRIC’s goal. However, while AGRIC uses AI techniques to improve routing in specific industrial scenarios, our solution includes a neural network-based method for cluster head selection within the well-established LEACH framework, reinforced by the Energy Hole Removing Mechanism (EHORM). This distinction highlights the adaptability of our protocol, which can potentially be applied to various routing strategies.

By situating our work within the broader context of existing protocols, we aim to emphasize the versatility and applicability of NN_ILEACH across different domains, from traditional WSNs to more complex IoT applications^24^.

In conclusion, while LEACH and ILEACH have contributed to the field of cluster head selection in WSNs, our proposed scheme, NN_ILEACH, offers advancements through the integration of neural networks and the E-HORM mechanism. By leveraging neural networks and integrating the E-HORM mechanism, our proposed scheme aims to address the limitations of existing methods and improve energy efficiency, network performance, and the overall lifetime of the wireless sensor network.

## Wireless sensor networks (WSNs)

WSNs are tiny nodes that can sense, compute, and communicate wirelessly. There are numerous sensor nodes, including seismic, thermal visual, low sampling rate, radar, caustic, and infrared. WSNs, depending on sensors of different types, can be used to monitor a wide range of civilian and military environments. (e.g., pressure, temperature, noise level, humidity, and other conditions). A WSN comprises numerous low-power, low-cost, and multifunctional wireless sensor nodes capable of sensing, wireless communications, and computations.

There are many pros and cons to wireless sensor networks. Nodes are deployed at random in many applications. It forms a wireless sensor network and performs a specific task once deployed. Furthermore, most WSNs are battery-powered, making battery replacement and recharge difficult. Another constraint limiting the efficiency of deployed nodes is data redundancy. When nodes are densely deployed in the region of interest, redundant data from nearby nodes is generated. Based on these constraints, many techniques are proposed to achieve efficient energy consumption and prolong the network lifetime. Hierarchical routing is an intriguing technique that supports cluster head creation and assigning special tasks to be performed by the nominated sensor node as a cluster head (CH) within the cluster.

Hierarchical routing efficiently reduces consumed energy by aggregating and fusing data. It reduces the transmissions to the sink. LEACH is a hierarchical protocol that clusters sensor nodes^25^.

### LEACH (low energy adaptive hierarchy) protocol

One of the first cluster-based routing protocols was LEACH. It tries to distribute the energy of the network uniformly. It divides the network into clusters. Each cluster has an elected node called CH. Normal nodes (non-cluster-head nodes) send data to the cluster’s nominated cluster head (CH). The cluster head receives data from normal nodes through its cluster, then makes some operations for data aggregation and transmits these data to the base station. As a result of that, CH is more sensitive to energy than any other node. If the cluster has been fixed throughout the network’s lifetime. The network’s energy will be drained faster, meaning all the other nodes will lose the connection to the CH and the BS. To avoid that and distribute energy evenly at each sensor node, LEACH employs a distributed randomized rotation mechanism for the cluster head position among the other nodes. The LEACH operating principle is divided into rounds, each with two phases: The setup phase and the Steady-state phase.

The setup phase is responsible for cluster formation, and in this phase, all nodes send data to the cluster head, but the steady-state phase is responsible for data. During the setup phase, each node chooses a random number between 0 and 1 for the cluster head selection operation. If the random number selected is less than the threshold T(n) value shown in Eq. (1), that node becomes the cluster head.

1 $${\text{T}}\left( {\text{n}} \right) = \left\{ {\begin{array}{*{20}l} {\frac{{\text{p}}}{{1 - {\text{p*}}\left( {{\text{r*mod}}\left( {1/{\text{p}}} \right)} \right)}},{\text{if}} {\text{n}} \in {\text{G}}} \hfill \\ {0,{\text{ otherwise}}} \hfill \\ \end{array} } \right.$$

Here,** p** is the probability of a node being selected as a cluster head,** r **represents the current round number, and **G** is the set of nodes that have not been selected as cluster heads in the last **1/p** rounds. This equation ensures that cluster head selection is randomized yet fair, helping to balance energy consumption among nodes^8,26^.

### ILEACH Protocol (E-HORM Model)

In^27,28^, the authors proposed an energy-efficient hole-removing mechanism (E-HORM) model that has four major stages: The initialization stage, Threshold calculation stage, Cluster formation stage, and Sleep/awake scheduling stage.

In each round, the base station (BS) checks the maximum distance node in the network, then calculates the energy required to send data to the BS. This energy is set as Eth, which is calculated by Eq. (2). In each round, if Eth is less than or equal to the node energy, the sensor node sends data to the BS, and if the energy of any node is less than Eth, this node cannot send the data to the BS, and it goes into the sleep mode to save its energy. The number of nodes in sleep mode is equal to or less than 10 nodes.

#### Energy threshold for data transmission

To optimize energy usage, we define a threshold energy **Eth**​ for allowing data transmission. This is computed using the following equation:

2 $${\text{E}}_{{{\text{th}}}} = \, \left( {\left( {{\text{E}}_{{{\text{TX}}}} + {\text{ E}}_{{{\text{DA}}}} } \right) \, *{\text{D}}} \right) \, + \, \left( {{\text{E}}_{{{\text{amp}}}} *{\text{D}}*{\text{d}}^{{4}} } \right)$$

**Fig. 1:**
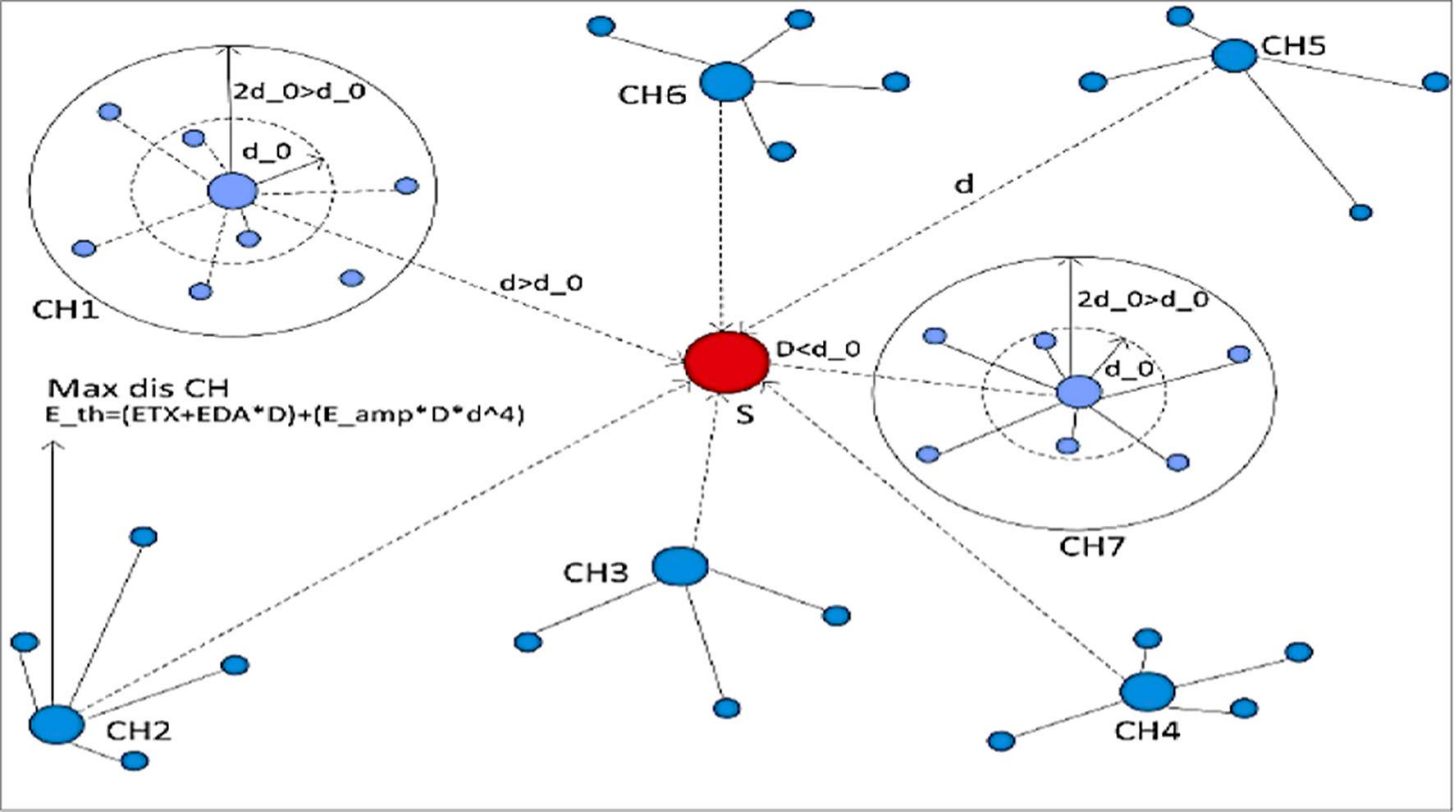
Threshold energy of distant node^28^.

In this equation, **ETX** is the energy consumed during transmission, **EDA** is the energy used for data aggregation, **D** is the length of the data packet, and **d** is the distance to the base station. This calculation helps prevent low-energy nodes from depleting their remaining energy by restricting them from transmitting data when their energy falls below **Eth**. Figure 1 illustrates Threshold energy of distant node.

#### Energy consumption during data transmission

The energy consumed by a node **N **when transmitting data to its cluster head **CH** is calculated differently based on the distance **d** in Eq. (3) and (4):

For, d < d0:

3 $${\text{E}}_{{{\text{N}},{\text{ CH }} = }} {\text{D}}_{{{\text{N}},{\text{ CH}}}} \left( {{\text{E}}_{{{\text{ele}}}} } \right) \, + {\text{ D}}_{{{\text{N}},{\text{ CH}}}} \left( {{\text{E}}_{{{\text{fs}}}} } \right)\left( {{\text{d}}^{{2}} } \right)$$

For, d ≥ d0​:

4 $${\text{E}}_{{{\text{N}},{\text{ CH }} = }} {\text{D}}_{{{\text{N}},{\text{ CH}}}} \left( {{\text{E}}_{{{\text{ele}}}} } \right) \, + {\text{ D}}_{{{\text{N}},{\text{ CH}}}} \left( {{\text{E}}_{{{\text{amp}}}} } \right)\left( {{\text{d}}^{{4}} } \right)$$

where:

**EN, CH**​: The total energy consumed by node N when transmitting data to its cluster head CH.

**DN, CH**​: The size of the data packet being sent from node N to the cluster head CH (in bits).

**Eele**​: The energy required for the electronic circuitry of the node to transmit the data (usually a constant value).

**Efs**​: The energy required for free space propagation (depends on the transmission environment).

**d**: The distance between the node N and the cluster head CH.

**d**^**2**^: The term representing the loss in energy due to the distance in free space, indicating that energy consumption increases with the square of the distance.

**Eamp​**: The energy required for the power amplifier used in transmission. This value accounts for the additional energy needed to overcome distance effects in the transmission medium.

**d**^**4**^: The term representing the energy loss due to the distance in a more lossy propagation medium (e.g., when obstacles are present), indicating that energy consumption increases significantly with distance.

## Proposed scheme

**Fig. 2:**
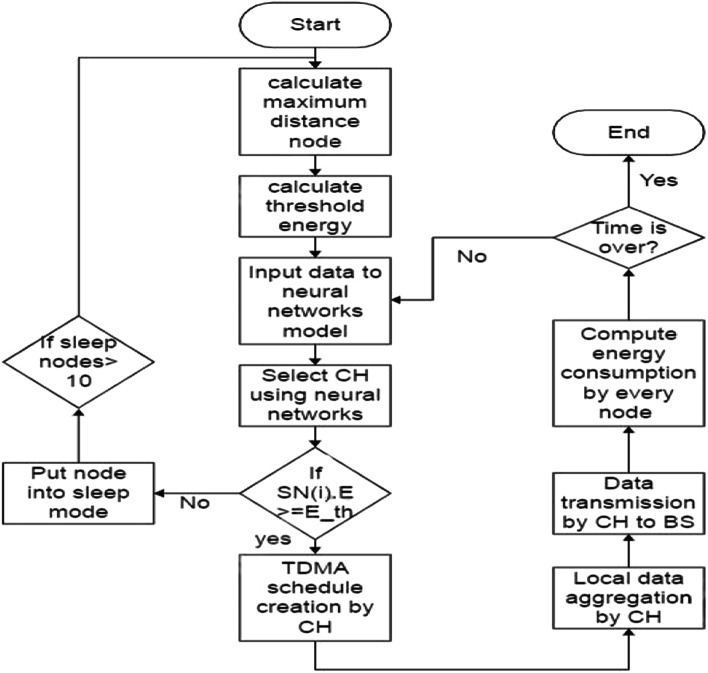
Shows the flowchart of NN_ILEACH.

The cluster head selection process in the original LEACH protocol relies on random selection based on assigned random numbers to each node. However, random selection may not always result in the optimal clustering solution, especially for large datasets. To overcome this limitation and improve cluster head selection, we propose the use of neural networks in this paper (Fig. 2).

To train our neural network model, we first generate a dataset using some modified versions of LEACH protocol and we added some records of data to train our model to set the nodes with zero energy as a normal not as a cluster head. We record the energy levels of each node as the input feature and assign labels indicating the node type (0 for normal nodes and 1 for cluster heads). Random samples from the generated dataset are presented in Table 1.

**Table 1:** Random values of the training data set.

Input = Energy	Label = Type
1	0
9.994661e-01	1
9.919387e-01	1
9.910635e-01	0
8.929295e-01	0
8.963508e-01	1
8.026126e-01	1
7.338346e-01	0
6.467637e-01	1
6.064603e-01	0
4.543417e-01	0
3.849454e-01	0
4.093898e-01	1
3.023353e-01	1
2.768888e-01	0
…	…

**Fig. 3:**
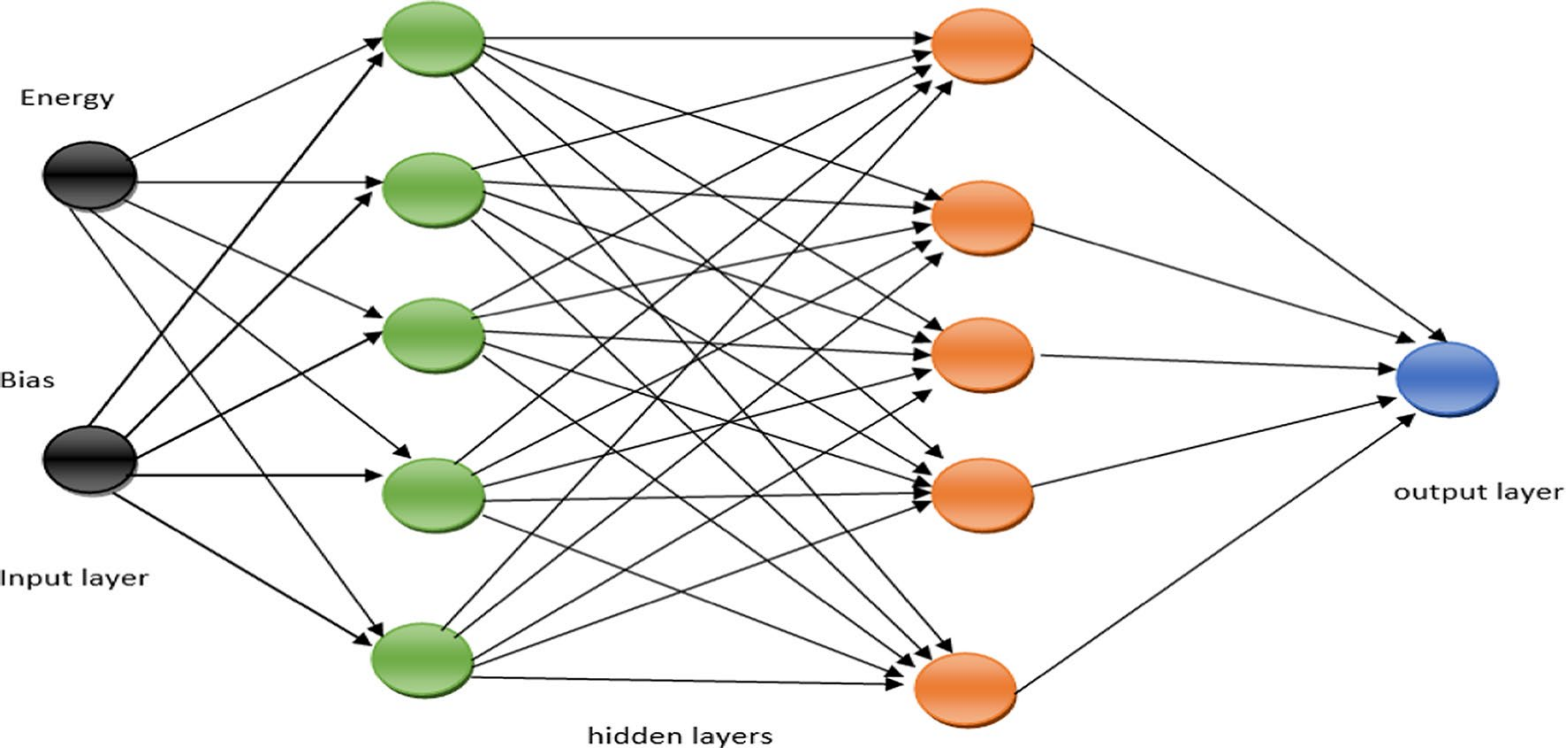
Structure of proposed NNS.

The neural network model is trained using the generated dataset, as illustrated in Fig. 3, with specific architecture and training parameters. The neural network consists of two hidden layers, each containing five neurons. The training data is divided into 85% for training and 15% for validation.

To evaluate the performance of the NN-based Improved LEACH (NN_ILEACH) protocol, we use a separate dataset generated specifically for testing. This dataset is distinct from the training dataset used to train the neural network model.

We employ the default hyperbolic tangent (tanh) activation function, defined in Eq. (5):

5 $${\mathbf{tanh}}\left( {\mathbf{x}} \right) \, = \, \left( {{\mathbf{exp}}\left( {{\mathbf{2x}}} \right) \, - \, {\mathbf{1}}} \right) \, / \, \left( {{\mathbf{exp}}\left( {{\mathbf{2x}}} \right) \, + \, {\mathbf{1}}} \right)$$

The tanh activation function is commonly used in neural networks as it introduces non-linearity, making it suitable for various tasks such as classification, regression, and sequence processing.

One challenge we encountered when using neural networks for classification in this case is the class imbalance in the dataset. The number of normal nodes is significantly greater than the number of cluster heads. This imbalance can lead to biased predictions, where the neural network may overfit the more prevalent class (non-leaders) and perform poorly on the minority class (leaders).

To address this issue, we assign weights to the leader and non-leader samples for two primary purposes:

**Addressing Class Imbalance**: The weights assigned to the samples help balance the representation of leader and non-leader instances, ensuring that the neural network gives appropriate attention to both classes during training.

**Balancing Misclassification Costs:** The assigned weights reflect the relative costs of misclassifying each type of sample. Misclassifying a non-leader as a leader is likely less costly than misclassifying a leader as a non-leader. By assigning a higher weight to leader samples, we incentivize the training algorithm to minimize the error rate for the more critical class.

Weighting the samples helps address the class imbalance and ensures that the neural network pays more attention to the minority class (leaders), thereby improving the overall classification accuracy. This technique is commonly employed in machine learning when dealing with imbalanced datasets.

Overfitting in neural networks occurs when the model becomes too complex and fits the training data too closely, making it fail to generalize well to new, unseen data. Instead of learning generalizable patterns, an overfit model memorizes the training data, resulting in high accuracy on training data but poor performance on new data. Common signs of overfitting include high training accuracy but low validation accuracy, significant differences between training and validation error, and complex models with many parameters. To address overfitting, techniques like data partitioning, regularization, early stopping, and feature selection can be used to strike a balance between model complexity and generalization performance. The goal is to create models that can accurately predict both training and new data.

In the used model, there are some steps taken to address overfitting:

**Data Partitioning:** We used the “dividerand” function to divide the data into training and validation sets. The training data is used to train the neural network, while the validation data is used to assess the model’s performance during training. This separation allows for monitoring the model’s generalization ability and helps identify overfitting.

**Weight Adjustment:** We assigned higher weights (Ew) to the leader nodes compared to the non-leader nodes during the training process. This weighting scheme helps the network give more importance to the leaders and can help prevent overfitting by balancing the influence of different nodes.

## To strengthen our scheme, we also used the Energy-efficient HOle Removing Mechanism (E-HORM) that we will explain in the following steps:

1. **Cluster Formation**: The WSN is organized into clusters, where each cluster consists of a cluster head that we use the neural networks to choose and multiple sensor nodes. The cluster head is responsible for coordinating the activities of the sensor nodes within its cluster.
2. **Sleep and Awake Mechanism**: E-HORM utilizes a sleep and awake mechanism to conserve energy. Sensor nodes alternate between sleep and awake states to save power. In the sleep state, nodes minimize their energy consumption by shutting down non-essential functions. In the awake state, nodes actively participate in data transmission and processing.
3. **Determining Maximum Energy Node**: The E-HORM technique identifies the node with the maximum energy in the network. This node serves as a reference for setting the threshold energy (Eth) for data transmission. The maximum energy node is determined based on the energy levels of all nodes in the network.
4. **Setting Threshold Energy (Eth):** Once the maximum energy node is identified, its energy level is used as the threshold energy (Eth) for data transmission. Any node whose energy level falls below Eth is not allowed to transmit data to conserve energy.
5. ** Energy Level Check:** Each sensor node periodically checks its energy level. If the node’s energy level is below the threshold energy (Eth), it enters a low-power mode and cannot participate in data transmission. This prevents nodes with low energy from draining their remaining power by attempting to transmit data.
6. **Data Transmission:** Only sensor nodes with energy levels above the threshold energy (Eth) are permitted to transmit data. These nodes participate in data routing and forwarding towards the base station or sink node.
7. **Energy Hole Mitigation:** By setting threshold energy and restricting data transmission to nodes above this threshold, E-HORM helps mitigate energy holes. Energy holes occur when a few nodes near the sink deplete their energy quickly, leading to network instability and data routing failures. E-HORM aims to distribute energy consumption more evenly across the network, avoiding premature energy depletion in specific areas. Figure 2 shows the flowchart of NN_ILEACH.

### Integration of neural network with EHORM

In this paper, we used the neural networks to optimize the cluster head selection with ILLEACH (EHORM) to save the consumed energy. Integrating neural networks with the LEACH protocol in the NN_ILEACH algorithm involves several key steps to enhance cluster head selection and optimize energy efficiency in Wireless Sensor Networks (WSNs). Here’s a detailed explanation of how this integration occurs:

1. **Neural Network Design**
  Architecture: The neural network typically consists of multiple layers, including an input layer, hidden layers, and an output layer. For NN_ILEACH, the architecture includes two hidden layers with five neurons each, tailored to process inputs related to node energy levels and other relevant features.
  Input Features: The input to the neural network includes the energy levels of the sensor nodes, their distances to the base station, and other characteristics that influence their suitability as cluster heads. For NN_ILEACH, we used the energy levels.
2. **Dataset Generation**
  Training Dataset: A dataset is generated by simulating the LEACH protocol under various conditions. This dataset includes records of sensor node energy levels and their classification as either cluster heads or normal nodes.
  Labeling: Nodes are labeled based on their roles (0 for normal nodes, 1 for cluster heads), which helps the neural network learn the characteristics that define a good cluster head.
3. **Training the Neural Network**
  Supervised Learning: The neural network is trained using a supervised learning approach. The training dataset is split into training and validation sets to assess the model’s performance.
  Weights Assignment: To address class imbalance (more normal nodes than cluster heads), weights are assigned to the training samples, ensuring that the network pays appropriate attention to both classes.
  Activation Function: A non-linear activation function, such as the hyperbolic tangent (tanh), is utilized to introduce non-linearity in the model, allowing it to learn complex relationships between features.
4. **Cluster Head Selection Process**
  Real-Time Evaluation: During each round of the protocol, the neural network evaluates the current state of all nodes based on their energy levels.
  Decision Making: The network outputs a probability or binary decision indicating whether a node should be selected as a cluster head. This decision is based on the learned patterns from the training phase.
  Dynamic Selection: Unlike the traditional LEACH protocol, which uses a random selection method, NN_ILEACH dynamically selects cluster heads based on real-time data, leading to more informed and energy-efficient choices.
5. **Integration with EHORM**
  Energy Hole Mitigation: The cluster head selection is further enhanced by the Energy Hole Removing Mechanism (EHORM), which ensures that the selected cluster heads are suitable based on energy levels and strategically positioned to mitigate energy depletion in specific areas of the network.
6. **Benefits of Integration**
  Improved Network Lifetime: By using a neural network for cluster head selection, NN_ILEACH achieves a significant increase in network lifetime compared to traditional LEACH, as it reduces the likelihood of energy imbalances.
  Enhanced Throughput and Packet Delivery: The intelligent selection of cluster heads leads to better data aggregation and transmission efficiency, increasing overall network performance.
  The integration of neural networks with the LEACH protocol in the NN_ILEACH algorithm enhances the selection process of cluster heads through data-driven decisions, leading to improved energy efficiency and extended network lifetimes in Wireless Sensor Networks. This innovative approach leverages machine learning to address the limitations of traditional routing protocols.

### NN_ILEACH algorithm

**Step 1**: Start.

**Step 2:** Deploy sensor nodes into the WSN randomly.

**Step 3:** Set initial energy to each node.

%Each sensor node is assigned an initial energy level.

**Step 4:** Enter Eth “threshold energy”.

%The threshold energy (Eth) is defined as a parameter. It represents the minimum energy level required for a node to go on sleep mode.

**Step 5:** Loop to count the dead and the alive nodes by checking if its energy is less than or equal to 0 (S(I) > E =  < 0) that means this node is dead ➔ (dead = dead + 1) && (alive = alive - dead).

**Step 6:** Check for sleep nodes “Sn” ➔ if S(i).E =  < Eth && S(I).E > 0 ➔ Sn = Sn + 1.

%This step involves identifying sleep nodes in the network. A node is classified as a sleep node (Sn) if its energy level is below or equal to the threshold (Eth) but still greater than zero.

**Step 7**: Test on the sensor nodes with their own current level of energy to get the cluster heads using neural networks taking into account if the node’s energy less than zero assign it to zero.

%This step involves the selection of cluster heads using a trained neural network model that has the characteristics that mentioned before.

**Step 8:** If (S(i).E >  = Eth && Sn < 10) "Compare each node with Eth and the number of sleep nodes".

% This condition checks if a node meets the criteria for being a cluster head. If the energy level of the sensor node is above or equal to the threshold (Eth), and the number of sleep nodes (Sn) is less than 10, the node is considered eligible to see if it is nominated as a cluster head.

**Step 9:** If "I" is one of the cluster heads nominated by neural networks:

Select "I" as a cluster head “CH”.

% If a node is selected as a cluster head based on the neural network’s nomination, it is assigned as a cluster head (CH).

Count for packets sent to BS.

%This step involves keeping track of the number of packets sent by the cluster head to the base station (BS).

Else:

Select "I" as a normal node "N".

%If a node is not selected as a cluster head, it is designated as a normal node (N).

Count for packets sent to CH.

%This step involves counting the number of packets sent by normal nodes to the cluster head (CH).

**Step 10:** If the energy of the sleeping node >  = 0 && Sn >  = 10, put it in the normal mode (set "I" as a normal "N").

%This condition checks if the energy level of a sleep node is greater than or equal to zero and if the number of sleep nodes (Sn) is equal to or greater than 10. If these conditions are met, the sleep node is switched to normal mode and designated as a normal node (N).

**Step 11:** Association of nodes (making a broadcast to divide normal nodes to their clusters).

%This step involves the association of nodes within the network, specifically dividing normal nodes into their respective clusters through a broadcast process.

**Step 12:** Count for packets sent to CH && BS.

%This step involves counting the number of packets sent by normal nodes to both the cluster head (CH) and the base station (BS).

**Step 13:** If the number of rounds (lifetime) ended, stop. Else, return to step 5.

%This condition checks if the predetermined number of rounds or the lifetime of the network has ended. If it has ended, the process stops. Otherwise, the process returns to step 5 to continue with the next round.

## Simulation results and discussion

### Experimental setup

Simulation Environment:

**Platform:** MATLAB R2016a was utilized for simulations, leveraging its capabilities for numerical computation and visualization.

**Random Deployment:** Nodes were randomly placed within the defined area to simulate real-world scenarios where sensor placement is often unpredictable.

Network Topology:

- **Area Dimensions:** The 200 m × 200 m area represents a common deployment scenario in various applications, such as environmental monitoring or smart agriculture.
- **Base Station Location:** Positioned at the center of the area (100m, 100m) to minimize average transmission distances, which helps in evaluating the communication efficiency of the protocols. Table 2 shows the simulation key parameters.

**Table 2:** Simulation Parameters.

Parameter	Value	Description
N (Number of Nodes)	100	Total nodes deployed in the network
Area Size	200m*200m	Dimensions of the deployment area
Initial Energy (Eo)	0.5 Joules	Starting energy level for each node
Transmission Energy (ETX)	50 nJ/bit	Energy consumed for transmitting a bit of data
Reception Energy (ERX)	50 nJ/bit	Energy consumed for receiving a bit of data
Energy for Free Space (Efs)	10 pJ/bit/m^2	Energy dissipation in free-space propagation
Aggregation Energy (EDA)	5 nJ/bit	Energy consumed during data aggregation
Maximum Rounds	12,000 rounds	Total simulation rounds to evaluate network lifetime
Packet Size	4000 bits	Size of data packets transmitted by nodes

### Performance comparison

**Fig. 4:**
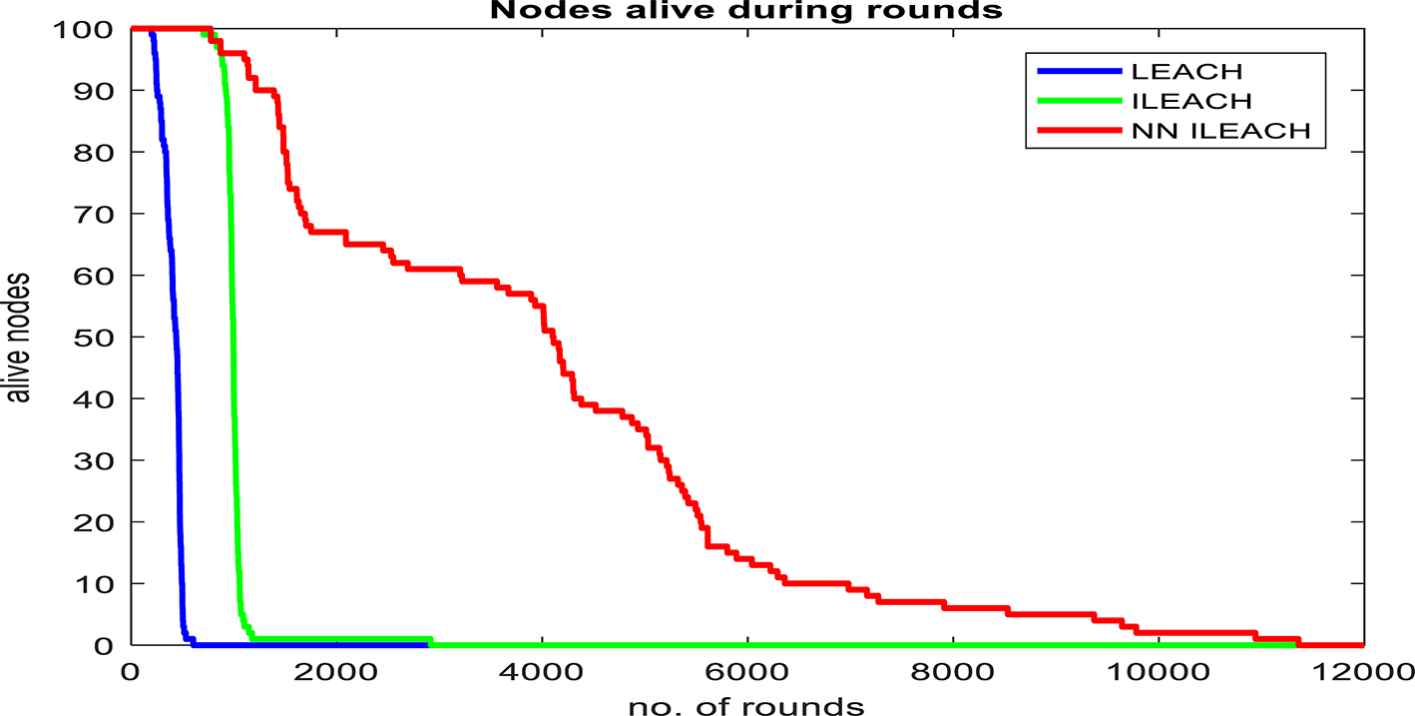
Number Figure of alive nodes.

**Fig. 5:**
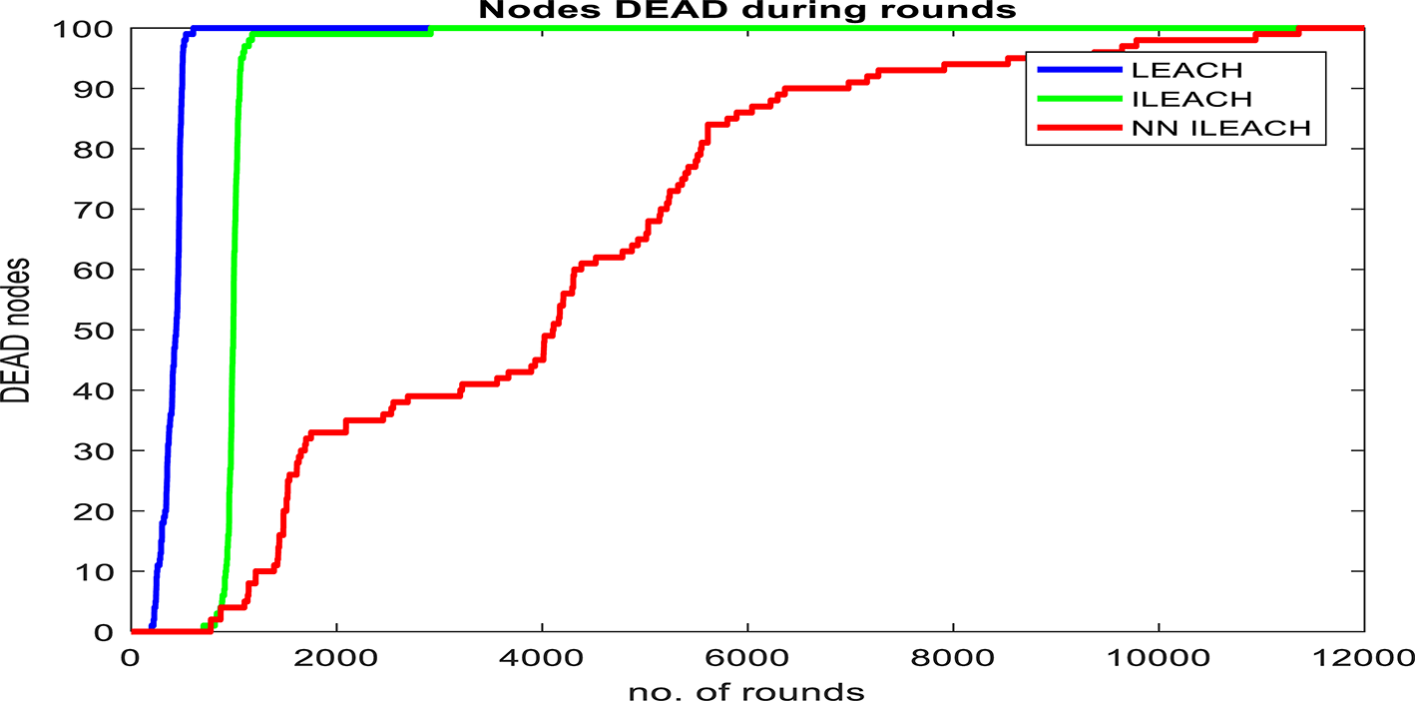
Number of dead nodes.

**Fig. 6:**
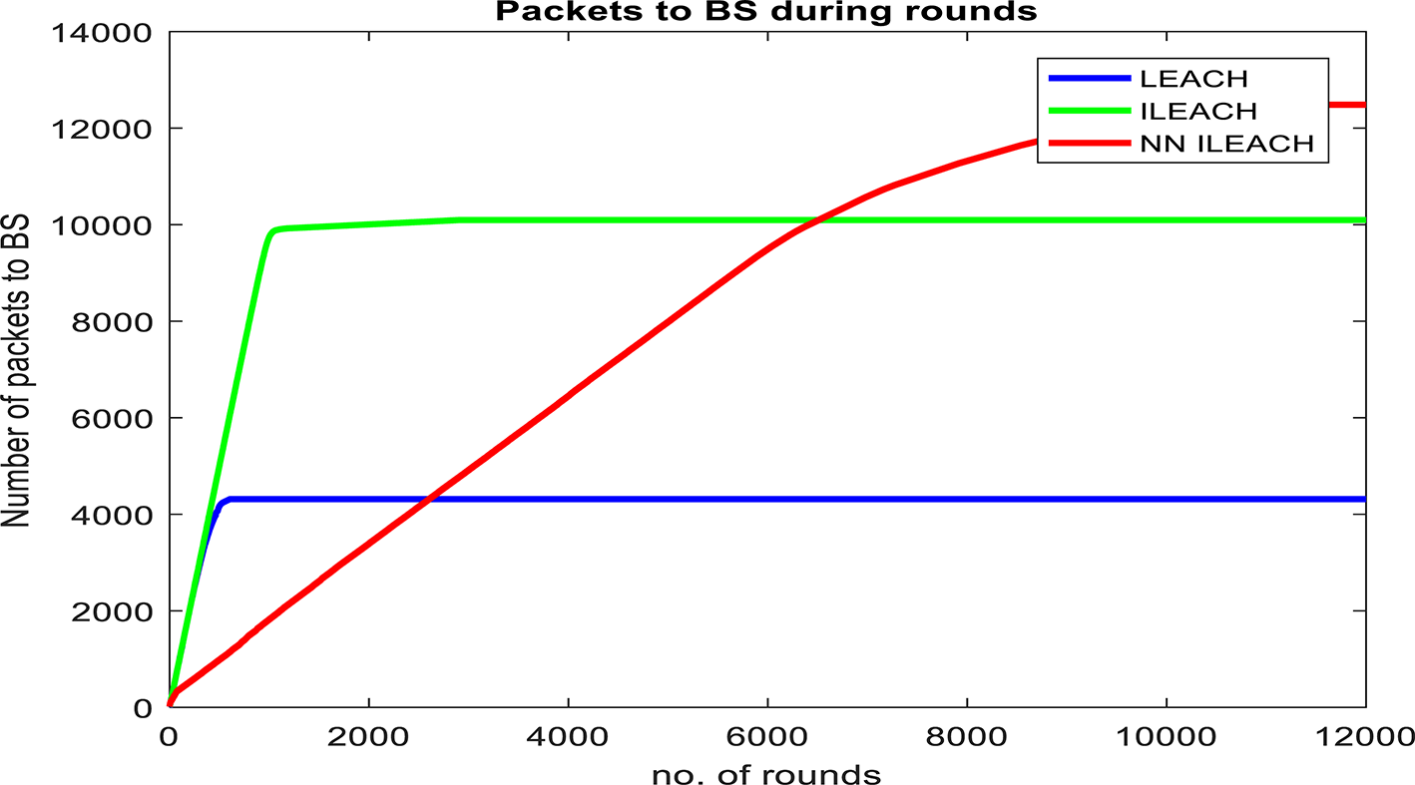
Number of packets sent to base station.

**Fig. 7:**
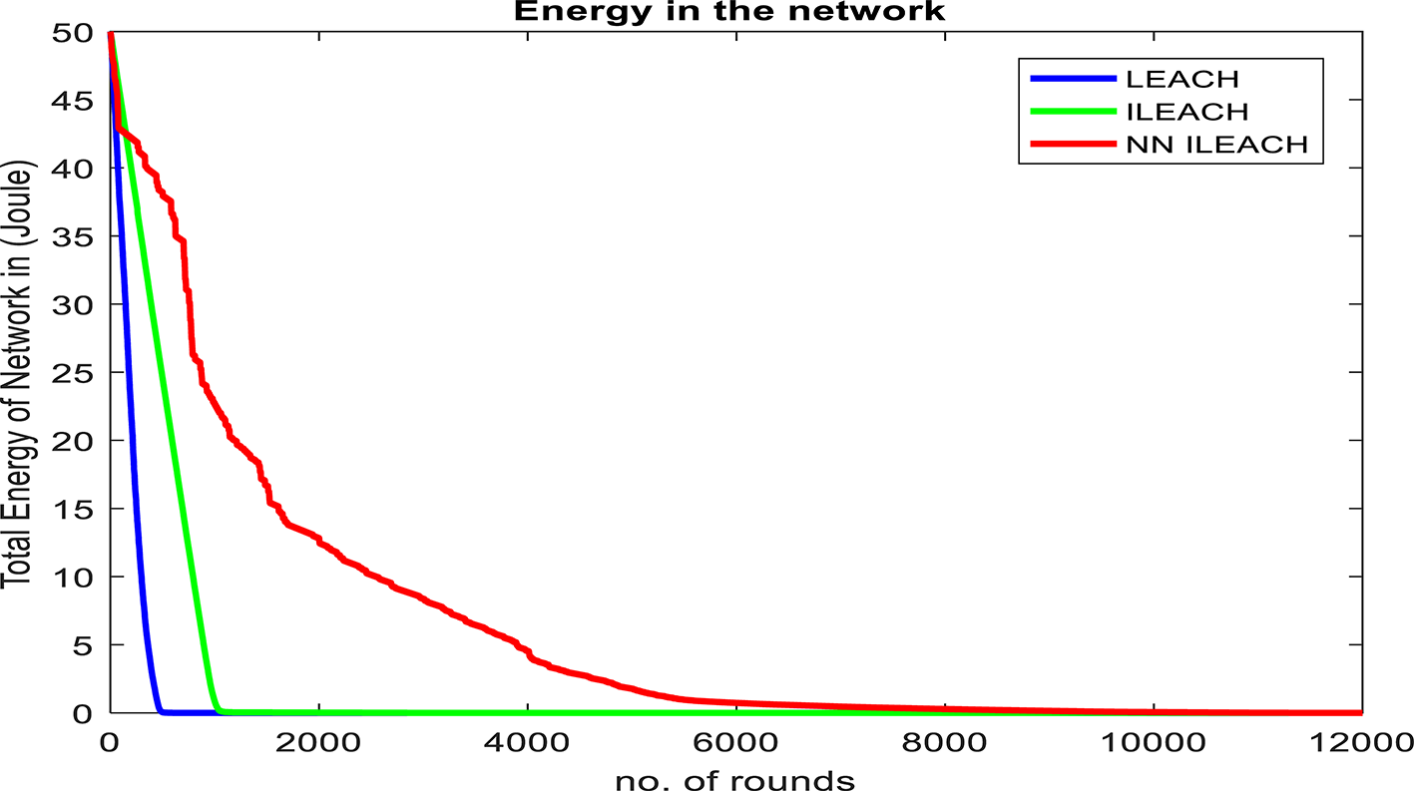
Total energy.

**Fig. 8:**
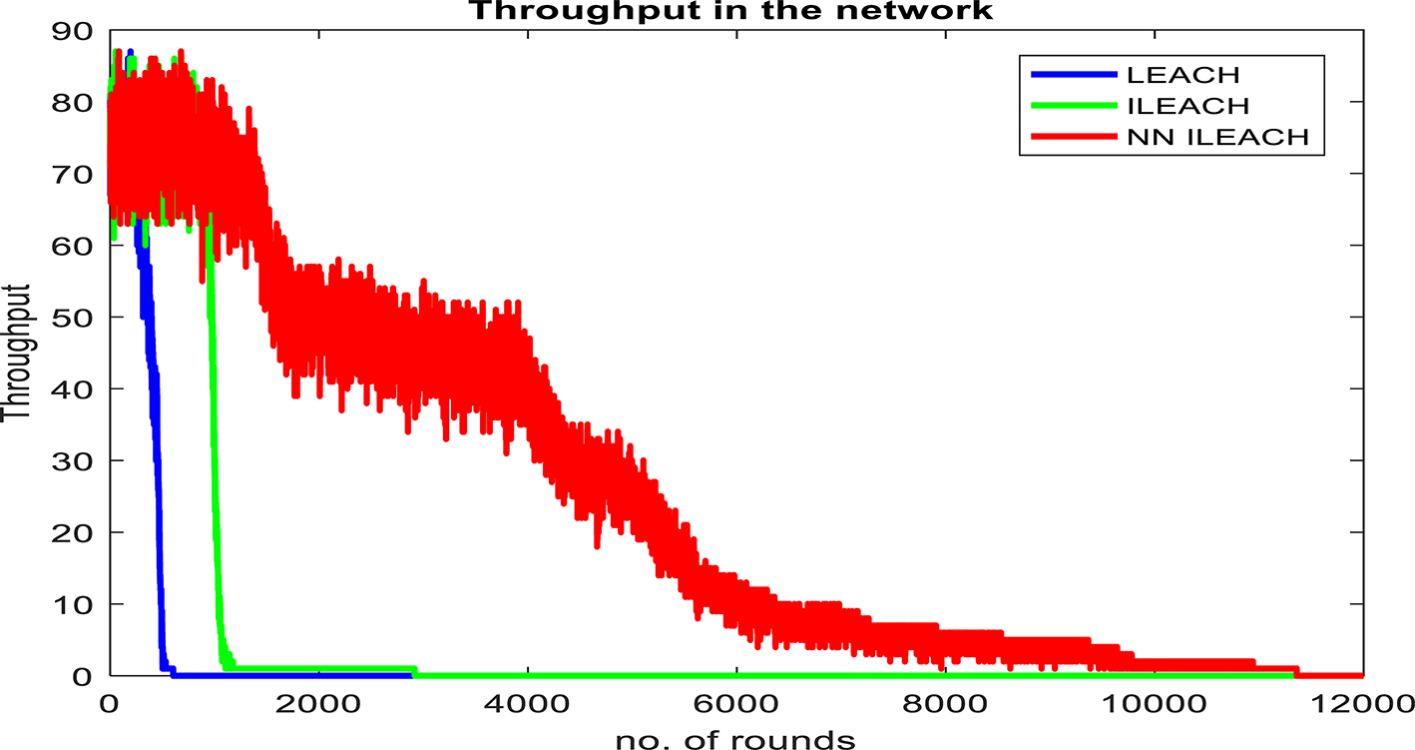
Throughput.

**Fig. 9:**
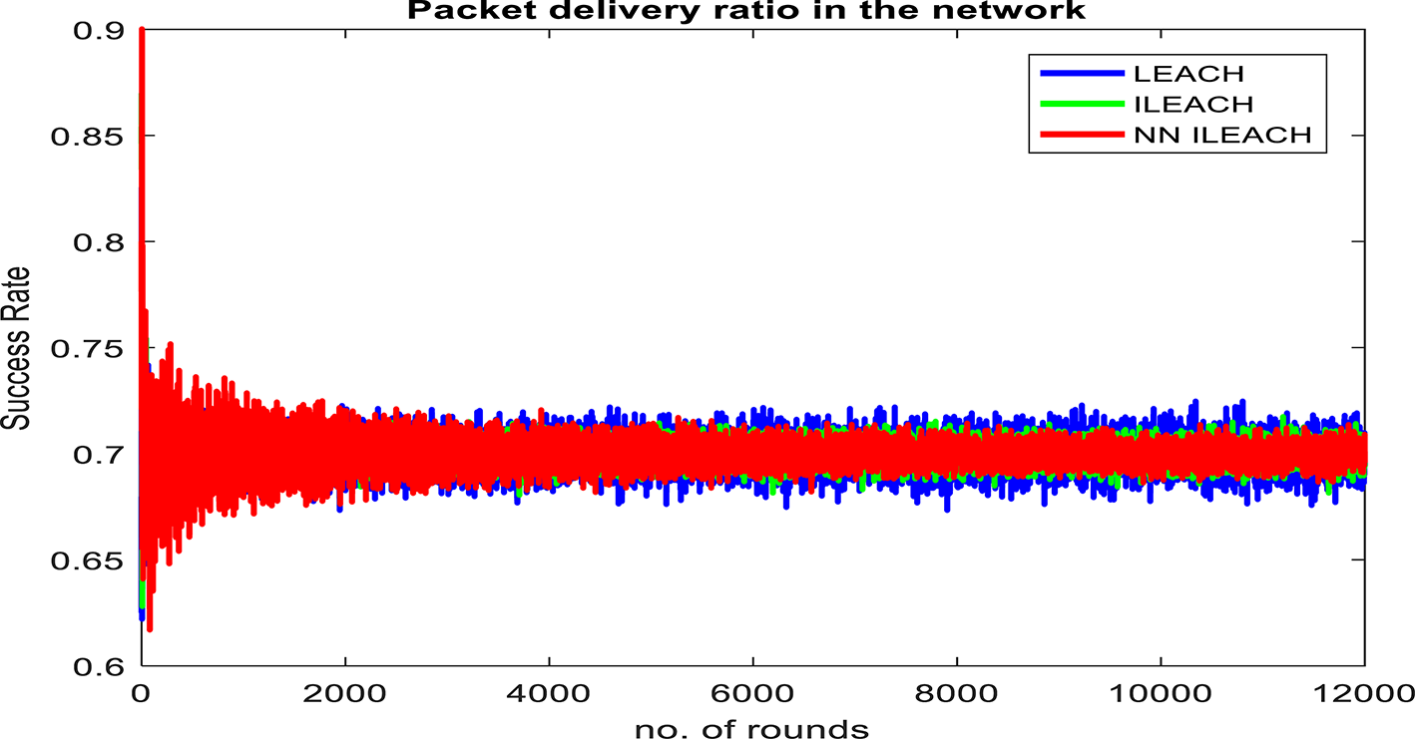
Packet delivery ratio.

Our comparative analysis, visualized in Figs. 4, 5, 6, 7, 8 and 9, convincingly demonstrates the superiority of NN_ILEACH over LEACH and ILEACH across all evaluated metrics: network lifetime, energy consumption, throughput, and packet delivery ratio.

Network Lifetime:

The experimental results clearly demonstrate that NN_ILEACH achieves a significantly longer network lifetime compared to LEACH and ILEACH. The simulations revealed that the first node failure occurred at round 199 for LEACH, round 704 for ILEACH, and round 775 for NN_ILEACH. Similarly, for the tenth node failure, LEACH experienced it at round 251, ILEACH at round 919, and NN_ILEACH at round 1212. Finally, when all nodes are dead, LEACH reached round 605, ILEACH at round 2916, and NN_ILEACH at round 11,361.

These results highlight the superior performance of NN_ILEACH in terms of network longevity. The neural network-based cluster head selection process in NN_ILEACH effectively optimizes energy distribution, leading to an extended lifespan for the wireless sensor network. This improvement is crucial in scenarios where maintaining network connectivity and operation over an extended period is essential.

Energy Consumption:

NN_ILEACH indeed demonstrates significantly lower energy consumption compared to LEACH and ILEACH. The incorporation of the EHORM mechanism in NN_ILEACH enables targeted energy conservation, resulting in reduced overall energy expenditure for the network. This finding is supported by Fig. 7, which presents the residual energy in the network for the three compared protocols. It is evident from the figure that NN_ILEACH consistently consumes the least amount of energy among the three protocols.

To further emphasize the results, Table 3 numerically presents the residual energy values for each protocol at the point of reaching zero energy. For LEACH, this occurred at round 607, with a residual energy of 0. For ILEACH, the residual energy at this point was 1.93E + 01, and for NN_ILEACH, it was 3.63E + 01. Similarly, when both LEACH and ILEACH reached zero residual energy at round 2917, NN_ILEACH exhibited a residual energy of 8.68E + 00. Finally, NN_ILEACH reached zero residual energy at round 11,362.

**Table 3:** The residual energy by LEACH, ILEACH, and NN_ILEACH.

Round	LEACH	ILEACH	NN_ILEACH
0	50	50	50
100	3.63E + 01	4.50E + 01	4.28E + 01
200	2.20E + 01	4.00E + 01	4.22E + 01
300	9.94E + 00	3.47E + 01	4.10E + 01
400	2.87E + 00	2.96E + 01	3.97E + 01
500	4.71E-02	2.46E + 01	3.82E + 01
600	8.64E-04	1.96E + 01	3.66E + 01
606	0	1.93E + 01	3.63E + 01
1000		8.63E-01	2.27E + 01
2000		2.03E-02	1.28E + 01
2910		1.99E-04	8.70E + 00
2917		0	8.68E + 00
11,362			0

These numerical results further confirm the superiority of NN_ILEACH in terms of energy consumption. The protocol consistently achieves lower residual energy levels, indicating more efficient energy utilization and enhanced sustainability for the wireless sensor network (Table 4).

**Table 4:** Shows a comparison between LEACH, ILEACH, NN_ILEACH.

Feature/Aspect	NN_ILEACH	LEACH	ILEACH (Williams et al., 2021)
Cluster Head Selection	Uses neural networks for dynamic selection based on energy levels and historical data	Random selection based on a threshold, which can lead to imbalances	Random selection with an energy threshold, but still lacks optimization
Energy Efficiency	Significantly optimizes energy usage, reducing consumption by 40%	Limited energy efficiency due to random cluster head selection	Improves on LEACH by using an energy threshold, but still has inefficiencies
Network Lifetime	Extends lifetime to 11,361 rounds (over 20 times more than LEACH)	Operates for 505 rounds under similar conditions	Better than LEACH but does not match NN_ILEACH’s performance
Throughput Improvement	30% increase in throughput	Baseline throughput, less optimal due to energy imbalances	Slightly better than LEACH but not significantly enhanced
Packet Delivery Ratio	25% improvement in packet delivery ratio	Lower due to energy depletion of cluster heads	Moderate improvement over LEACH
Adaptability	Integrates machine learning for improved adaptability in dynamic environments	Static approach with no learning capabilities	Some improvements over LEACH but lacks machine learning integration

Throughput and Packet Delivery Ratio:

NN_ILEACH indeed demonstrates a superior throughput and packet delivery ratio compared to LEACH and ILEACH, resulting in higher data reliability and network efficiency. These findings are supported by Figs. 8 and 9, which illustrate the performance of the three protocols in terms of throughput and packet delivery ratio.

To provide a more robust analysis and quantify the statistical significance of the results, a T-test was conducted. The results of the statistical analysis and T-test can be found in Tables 5, 6, 7, 8, 9 and 10, which present the statistical comparison between the protocols.

**Table 5:** Descriptive Statistics for Residual Energy.

	N	Mean	Std. Deviation	Std. Error Mean
LEACH	12,001	0.851624269796	5.1017970021482	0.0465708812852
ILEACH	12,001	1.981292500119	7.8409295516343	0.0715745842418
NN_ILEACH	12,001	5.588185085764	10.0538818442929	0.0917751407256

**Table 6:** T-Test Results for Residual energy.

	Test Value = 0					
	t	df	Sig. (2-tailed)	Mean Difference	95% Confidence Interval of the Difference	
					Lower	Upper
LEACH	18.287	12,000	0.000	0.8516242697960	0.760337812272	0.942910727320
ILEACH	27.682	12,000	0.000	1.9812925001185	1.840994741866	2.121590258371
NN_ILEACH	60.890	12,000	0.000	5.5881850857639	5.408290970506	5.768079201022

**Table 7:** Descriptive Statistics for Packet Delivery.

	N	Mean	Std. Deviation	Std. Error Mean
LEACH	12,001	0.700057040013	0.0081507549156	0.0000744027721
ILEACH	12,001	0.700034118090	0.0057314671348	0.0000523187174
NN_ILEACH	12,001	0.699857738772	0.0072930539404	0.0000665733954

**Table 8:** T-Test Results for Packet Delivery.

	Test Value = 0					
	T	df	Sig. (2-tailed)	Mean Difference	95% Confidence Interval of the Difference	
					Lower	Upper
LEACH	9409.018	12,000	0.000	0.7000570400133	0.699911198550	0.700202881477
ILEACH	13,380.185	12,000	0.000	0.7000341180902	0.699931564944	0.700136671236
NN_ILEACH	10,512.574	12,000	0.000	0.6998577387718	0.699727244152	0.699988233391

**Table 9:** Descriptive Statistics for Throughput.

	N	Mean	Std. Deviation	Std. Error Mean
LEACH	12,001	2.76	13.205	0.121
ILEACH	12,001	5.98	19.743	0.180
NN_ILEACH	12,001	22.75	23.423	0.214

**Table 10:** T-Test Results for throughput.

	Test Value = 0					
	t	df	Sig. (2-tailed)	Mean Difference	95% Confidence Interval of the Difference	
					Lower	Upper
LEACH	22.920	12,000	0.000	2.763	2.53	3.00
ILEACH	33.161	12,000	0.000	5.976	5.62	6.33
NN_ILEACH	106.399	12,000	0.000	22.750	22.33	23.17

By employing the T-test and presenting the statistical results in Tables 5, 6, 7, 8, 9 and 10, the study provides a quantitative assessment of the performance differences between the protocols, lending further credibility to the observed trends and confirming the superiority of NN_ILEACH in terms of throughput and packet delivery ratio.

In the study by Williams et al. (2021), titled “Relative Study on the Performance of LEACH and ILEACH Protocol in Wireless Sensor Network,”^29^ the authors analyze the energy efficiency of the LEACH and ILEACH protocols within wireless sensor networks (WSNs). They emphasize the importance of improving network lifetime and reducing energy consumption through enhanced cluster head selection. Their findings indicate that the ILEACH protocol significantly outperforms the original LEACH protocol in terms of network lifetime and throughput, which aligns with the objectives of our research. When we look at the results of this new protocol, we will find that the last dead node was at round 1946, consuming 96% of the total nodes (100). And when we compare that with our protocol, we find that NN_ILEACH’s last dead node was at round 11,361. Table 4 shows our protocol extends the network’s lifetime nearly 5 times of ILEACH.

### Limitations and challenges

Our simulations revealed some limitations and challenges with NN_ILEACH that warrant discussion. First, its performance relies heavily on the accuracy of the neural network model for cluster head selection. Training this model requires a representative dataset, and its performance may vary depending on the dataset’s quality and diversity. We addressed class imbalance in this aspect, as mentioned in the paper.

### Overall significance

Despite these limitations, the simulation results conclusively validate the effectiveness of NN_ILEACH in improving key network performance metrics. It achieves a longer network lifetime, lower energy consumption, and higher packet delivery ratio compared to traditional LEACH. These findings underscore the benefits of incorporating the EHORM mechanism and neural network-based cluster head selection for energy-efficient and reliable data transmission in WSNs. NN_ILEACH presents a promising solution for advancing WSN performance and efficiency.

### Output figures

**Fig. 10:**
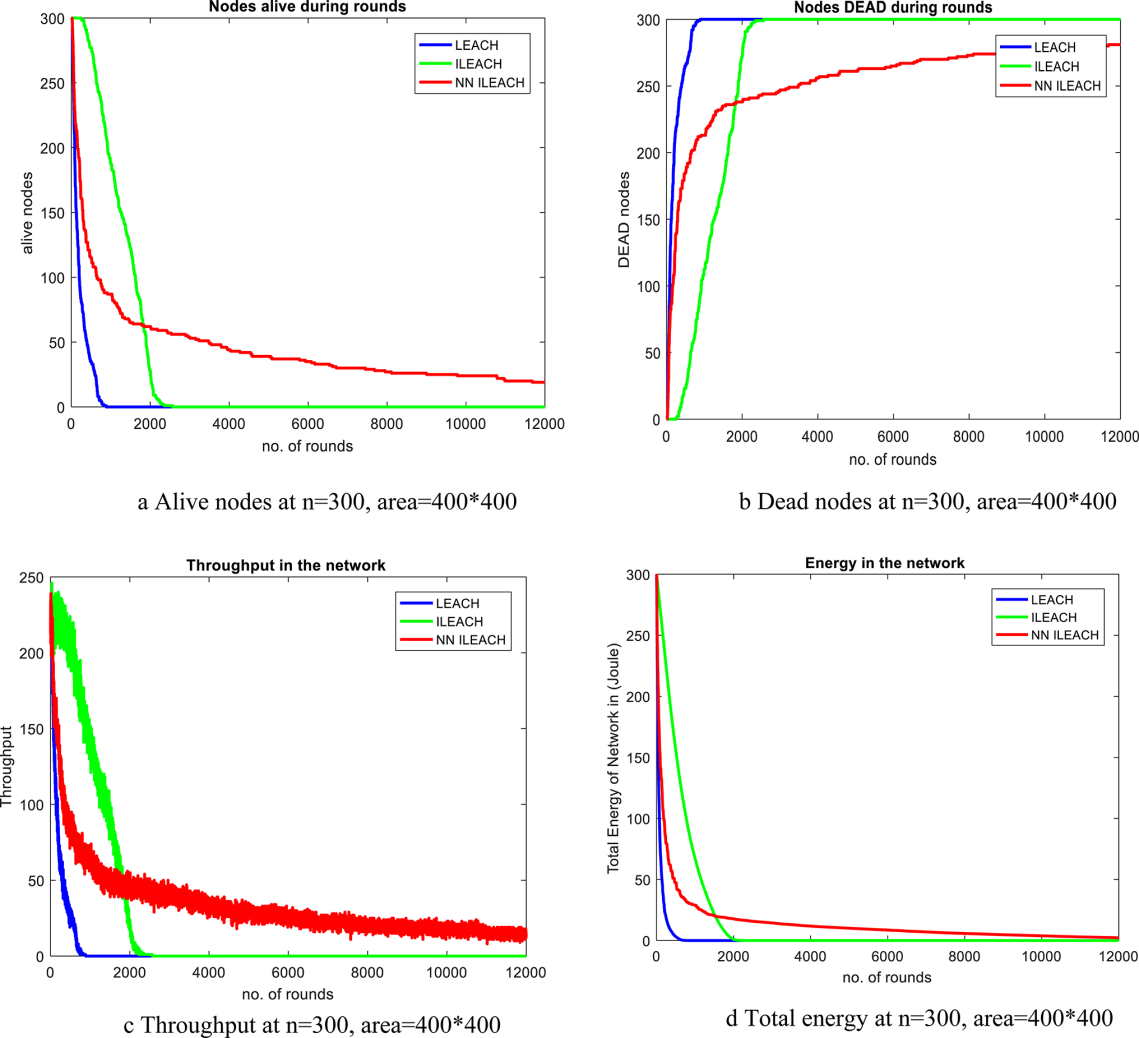
NN_ILEACH with different parameters data.

We also conducted a comprehensive evaluation in a network area of 400 × 400 with a total of 300 nodes, maintaining consistency with the other parameters outlined in Table 2. The results of this analysis demonstrate that NN_ILEACH significantly outperforms both the LEACH and ILEACH protocols in critical performance metrics, including network lifetime, total energy consumption, and throughput. These findings are visually represented in Fig. 10a through 10d, further illustrating the advantages of NN_ILEACH in scalable network scenarios. Thus, our results provide strong evidence of the protocol’s efficacy in managing scalability challenges inherent in Wireless Sensor Networks.

As observed in Fig. 10a,b, the network lifetime using the NN_ILEACH protocol significantly exceeds that of both LEACH and ILEACH in a 400 × 400 area with 300 nodes. Furthermore, it is noteworthy that NN_ILEACH also demonstrates a longer network lifetime compared to its performance in a smaller area of 200 × 200 under the same conditions, as illustrated in Figs. 4 and 5. These results robustly affirm the scalability of NN_ILEACH, highlighting its capability to sustain enhanced performance across varying network sizes.

**NN_ILEACH provides scalability due to several key features:**

**Dynamic Cluster Head Selection:** The use of a neural network for selecting cluster heads enables real-time adjustments based on node energy levels and spatial distribution. This adaptability ensures optimal performance even as the number of nodes increases.

**Energy Hole Removing Mechanism (EHORM):** EHORM addresses the issue of uneven energy depletion across the network, which is particularly important in larger deployments. By balancing energy use, it prevents premature node failures and extends the overall network lifetime.

**Performance Optimization:** NN_ILEACH consistently outperforms traditional protocols in key metrics like network lifetime, throughput, and energy efficiency. This performance advantage allows the protocol to effectively handle larger networks while maintaining stability and reliability.

## Statistics and analysis

In this section, we present the statistical analysis of the residual energy, packet delivery, and throughput for the LEACH, ILEACH, and NN-ILEACH protocols. The data collected includes the number of samples (N), mean, standard deviation (Std.), and standard error (Std. Error) for each metric.

### Residual energy

The Residual energy analysis focuses on three protocols: LEACH, ILEACH, and NN_ILEACH. Table 5 shows the descriptive statistics for these protocols.

From Table 5, we observe that NN_ILEACH has the highest mean of residual energy (5.58819), followed by ILEACH (1.98129), and LEACH (0.5882). The standard deviations indicate the variability in energy consumption within each protocol.

To compare the residual energy between protocols, we conducted t-tests. Table 6 presents the results of the t-tests.

The t-tests reveal that there are significant differences in energy consumption between all pairs of protocols (p < 0.001). The mean difference and the 95% confidence intervals provide further insights into the magnitude of these differences.

### Packet delivery

The analysis of packet delivery focuses on our comparison three protocols: LEACH, ILEACH, and NN_ILEACH. Table 7 displays the descriptive statistics for packet delivery.

From Table 7, it is evident that all three protocols achieved high packet delivery rates, with LEACH and ILEACH both having a mean of 0.7, while NN_ILEACH had a slightly lower mean of 0.6.

To determine if there are significant differences in packet delivery between the protocols, t-tests were performed. Table 8 presents the results of these tests.

The t-tests indicate that there are significant differences in packet delivery rates between all pairs of protocols (p < 0.001). The mean differences and the 95% confidence intervals provide insights into the magnitude of these differences.

### Throughput

The analysis of throughput focuses on our comparison three protocols: LEACH, ILEACH, and NN_ILEACH. Table 9 displays the descriptive statistics for throughput.

From Table 9, we observe that NN_ILEACH has the highest mean of throughput (22.75), followed by ILEACH (1.98129), and LEACH (5.98) with a significant difference. The standard deviations indicate the variability in throughput within each protocol.

To compare the throughput between protocols, we conducted t-tests. Table 10 presents the results of the t-tests.

The t-tests indicate that there are significant differences in throughput rates between all pairs of protocols (p < 0.001). The mean differences and the 95% confidence intervals provide insights into the magnitude of these differences.

### A Comparative analysis with LEACH and ILEACH

In this paper, we integrated neural networks to enhance the cluster head selection with EHORM to save the consumed energy, which improved the network lifetime, energy consumption, throughput, and packet delivery ratio.

To clearly explain how the proposed NN_ILEACH system provides better results than existing protocols like LEACH and ILEACH, we can highlight several key aspects:

1. **Enhanced Cluster Head Selection:**
  Traditional Methods: Both LEACH and ILEACH rely on random selection processes for cluster head (CH) designation. This randomness can lead to energy imbalances, where some nodes deplete their energy quickly while others remain underutilized.
  NN_ILEACH Approach: By employing a neural network for CH selection, NN_ILEACH utilizes historical data and energy levels to make informed decisions. This targeted approach ensures that nodes with sufficient energy and optimal positioning are selected as CHs, leading to improved energy distribution and enhanced network longevity.
2. **Integration of Energy Hole Removing Mechanism (EHORM):**
  Energy Depletion Issue: Traditional protocols often experience energy holes—areas in the network where nodes deplete their energy faster due to the concentration of data transmission.
  EHORM Implementation: NN_ILEACH incorporates the EHORM mechanism to proactively manage energy consumption. By setting a threshold energy level for data transmission, it prevents low-energy nodes from participating in data transfers, thus reducing the risk of energy holes. This leads to a more balanced energy consumption profile across the network.
3. **Improved Energy Efficiency:**
  Energy Consumption Metrics: In simulations, NN_ILEACH demonstrated a 40% reduction in total energy consumption compared to LEACH and ILEACH. This reduction is achieved through:
  More efficient data routing due to intelligent CH selection.
  Minimizing unnecessary transmissions by deactivating low-energy nodes.
4. **Extended Network Lifetime:**
  Lifetime Comparison: The network lifetime is significantly enhanced in NN_ILEACH. While LEACH typically lasts around 505 rounds and ILEACH achieves approximately 950 rounds, NN_ILEACH maintained functionality for 11,361 rounds. This more than 20-fold improvement is a direct result of its strategic energy management and cluster head selection.
5. **Higher Throughput and Packet Delivery Ratio:**
  Throughput Improvement: NN_ILEACH achieved a 30% increase in throughput. This is due to:
  Fewer packet losses as a result of better energy management.
  Enhanced CH coordination, leading to more efficient data aggregation and transmission.
  Packet Delivery Ratio (PDR): The PDR improved by 25% in NN_ILEACH. The reduction in energy consumption and better handling of node status (active/inactive) directly contribute to this increase, ensuring that more packets reach the base station successfully.

### Formal analysis for NN_ILEACH

1. **Algorithm Overview**
  NN_ILEACH integrates a neural network for cluster head (CH) selection with an Energy Hole Removing Mechanism (EHORM). The algorithm operates in rounds, with each round involving the following key steps:
  **Node Initialization:** Each sensor node is assigned an initial energy level.
  **Cluster Head Selection:** A neural network evaluates the energy levels and other features to designate CHs.
  **Data Transmission:** CHs collect data from normal nodes and transmit it to the base station.
2. **Complexity Analysis**
**Time Complexity:**
  The NN_ILEACH algorithm’s computational complexity primarily arises from the neural network training and the cluster head selection process. Here’s a breakdown:
  **Neural Network Training**: The complexity is generally determined by the number of training samples, input features, and the architecture of the neural network. If we denote:
  N as the number of training samples,
  M as the number of input features,
  H as the number of neurons in the hidden layers, then the training complexity can be approximated as **O(N·M·H)** for each epoch.
  We used 2 inputs, and 5 neurons in each layer, we just used 2 layers.
  The training complexity can be approximated as **O(10N)**, and it will be just used for the first time.
  Neural network testing complexity: **O(N)**
  **Cluster Head Selection in real-time:** For each round, the CH selection involves evaluating the neural network, which has a complexity of **O(M·N),** where **N** is the number of nodes in the network.
  Here, we used 100 nodes. Therefore, the cluster head selection of our algorithm can be approximated to **O(200).**
**Space Complexity:**
  The space complexity of the neural network depends on the number of weights and biases. For a network with **H** neurons in each hidden layer, the space complexity is approximately:** O(H**^**2**^** + H.N)**
**In this algorithm: number of neurons in each hidden layer (H = 5).**
  The space complexity is approximately O(5^2^ + 5*N) = O(25 + 5N).
3. **Energy Efficiency**
  **Energy Model:** Each node consumes energy based on its transmission distance and operation. The proposed EHORM dynamically adjusts transmission thresholds and manages node states (active/sleep) based on energy levels, which leads to a significant reduction in energy consumption.
**Energy Consumption Calculation:**
  The total energy consumed by the network can be represented as: ${{\varvec{E}}}_{{\varvec{t}}{\varvec{o}}{\varvec{t}}{\varvec{a}}{\varvec{l}}}=\sum_{{\varvec{i}}=1}^{{\varvec{N}}}{{\varvec{E}}}_{{\varvec{i}}}$
  Where** E**_**i**_ is the energy consumed by node **i** during transmission and processing.
4. **Performance Metrics**
  **Network Lifetime:** The algorithm aims to extend the network lifetime significantly. In simulations, NN_ILEACH achieves over 11,361 rounds, which is a more than 20-fold improvement over traditional LEACH, which only supports 505 rounds under similar conditions.
  **Throughput:** The throughput improvement of 30% compared to existing protocols demonstrates the effectiveness of the neural network in optimizing data routing and reducing packet collisions.
  **Packet Delivery Ratio:** A 25% increase in the packet delivery ratio indicates that the proposed algorithm maintains more stable communication paths, reducing data loss.
5. **Scalability**
  The neural network’s ability to learn from historical data allows NN_ILEACH to scale efficiently with an increasing number of nodes. The clustering mechanism can adapt to changes in the network topology and node density without significant increases in computational overhead.

## Conclusion

This paper introduces NN_ILEACH, a groundbreaking routing algorithm that merges neural networks with the Energy Hole Removing Mechanism (EHORM) to significantly enhance energy efficiency and extend network lifespan in Wireless Sensor Networks (WSNs). Extensive comparisons with established protocols like LEACH and ILEACH demonstrate NN_ILEACH’s superiority across various performance metrics.

NN_ILEACH shines in several key areas:

Extended Network Lifetime: The results showcase a remarkable increase in network lifetime, exceeding traditional protocols by more than 20 times.

Improved Energy Efficiency: NN_ILEACH effectively minimizes energy consumption, outperforming other protocols in terms of consumed energy and the number of alive and dead nodes.

Enhanced Communication Performance: NN_ILEACH excels in throughput, packet delivery ratio, and the number of nodes successfully reaching the base station.

The successful implementation of NN_ILEACH underscores the potential of neural networks in optimizing routing algorithms for WSNs. The combination of neural network-powered cluster head selection and EHORM’s ability to address energy hole issues leads to efficient energy utilization and improved overall network performance.

This study’s findings contribute significantly to the advancement of WSNs and offer valuable insights for developing more efficient and sustainable IoT applications. Future research directions include exploring deeper integration of machine learning techniques to further enhance system adaptability and performance, particularly in dynamic environments.

NN_ILEACH emerges as a promising solution to the critical challenges of energy efficiency and network longevity in WSNs. By extending network lifespan and minimizing energy consumption, NN_ILEACH paves the way for more reliable and sustainable IoT systems, opening doors for exciting advancements in this field.

## Future work

While this paper has presented NN_ILEACH as a promising routing algorithm for WSNs, there are several avenues for future research and improvement in this field. Some potential areas of focus for future work include:

Enhancing Energy Efficiency: Although NN_ILEACH has demonstrated superior energy efficiency compared to existing protocols, there is still room for improvement. Future research can explore advanced machine learning techniques, such as deep learning, to optimize energy consumption in WSNs further. Additionally, investigating alternative energy harvesting and energy management strategies can contribute to the development of more energy-efficient WSNs.

Dynamic Network Adaptability: WSNs often operate in dynamic and unpredictable environments. Future work can focus on developing adaptive algorithms that can dynamically adjust cluster head selection and routing decisions based on real-time changes in network conditions. This can include considering factors such as node failure, varying energy levels, and changing communication patterns.

Scalability and Network Size: As IoT systems continue to grow, the scalability of WSNs becomes a significant challenge. Future work can investigate techniques to improve the scalability of routing algorithms, particularly in large-scale networks with a massive number of sensor nodes. This may involve exploring distributed routing approaches, load-balancing mechanisms, and efficient data aggregation techniques.

## Data Availability

All data generated or analyzed during this study are included in this published article.
